# Congenital Epulis: A Case Report and Estimation of Incidence

**DOI:** 10.1155/2009/508780

**Published:** 2009-11-19

**Authors:** David Bosanquet, Graham Roblin

**Affiliations:** Department of Otorhinolaryngology, University Hospital of Wales, Cardiff CF14 4XW, UK

## Abstract

Congenital Epulis, also known as Neumann's tumour, is a rare congenital growth affecting the gingival mucosa of neonates. It is benign condition, seen more frequently in females, with multiple Epuli occurring in only 10% of cases. The cause and origin of Congenital Epulis remains unclear. In this article we present a case report of an otherwise healthy female neonate with two Congenital Epuli arising from the upper and lower gingival margin, which were successfully treated with surgical excision. We also present a review of the literature and an estimation of the incidence of Congenital Epulis based on our institutions figures, of 0.0006% (upper 95% confidence interval: 0.0035%).

## 1. Introduction

Congenital Epulis (also known as Congenital Gingival Granular Cell Tumour) is a rare benign congenital growth of the newborn. It was first described in 1871 by Neumann [1], hence the alternative name is Newmanns' Tumour. It usually presents at birth with an obvious mass arising from the gingival mucosa of the maxilla or mandible [2]. There is a marked female preponderance of 8:1. Multiple lesions are rare, occurring in only 10% of all case reports. The size of the mass varies from a few millimetres to 9 cm in diameter [3]. They can interfere with feeding and respiration. The recommended treatment is surgical excision under local or general anaesthetic, although spontaneous regression has been reported. There are no reports of recurrence, even if incomplete margins are excised, malignant change, or future disruption to teeth or gums [4].

## 2. Case Report

An otherwise healthy 1-day-old girl was referred to a large teaching hospital in Cardiff for diagnosis and treatment of two large masses protruding from her mouth. The baby had normal antenatal scans at weeks 12 and 20, and pregnancy had been unremarkable, other than mother being Group B Streptococcus positive from a high vaginal swab. Mother was fit and well gravida 2 para 1, with no drug history or family history of note. Baby was born at term plus eight days weighing 3.85 kg, pink and breathing spontaneously (Apgar: 9-10).

On examination there were two fleshy, pedunculated masses arising from the upper and lower alveolar ridges measuring 4 × 3 × 3 cm just to the right of the midline. There were no respiratory difficulties. A nasogastric tube was passed due to concerns over feeding. At that time a differential diagnosis of Congenital Epulis, Haemangiomas, and Teramomas was made.

**Figure 1:**
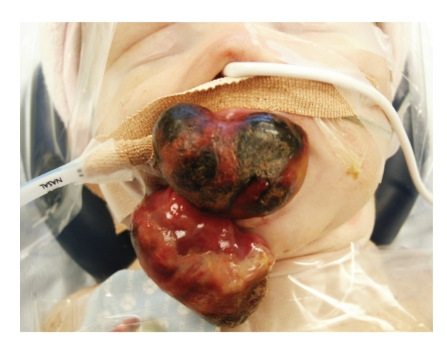
Intraoperative picture showing two large fleshy pedunculated masses arising from the upper and lower alveolar ridges.

**Figure 2:**
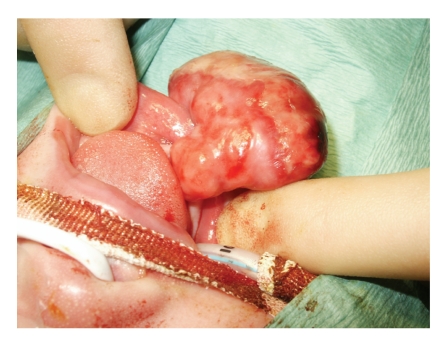
Intraoperative view showing the pedunculated nature of the mass arising from the lower alveolar ridge. The upper mass has been removed.

She was booked for excision of these masses under general anaesthesia (Figure 1). Both masses were removed with an eliptical insion to the peduncles (Figure 2). Hameostasis was with diathermy. There was minimal blood loss. Postoperative recovery was uneventful. The child was breastfeeding the day after surgery, and discharged home the following day.

**Figure 3:**
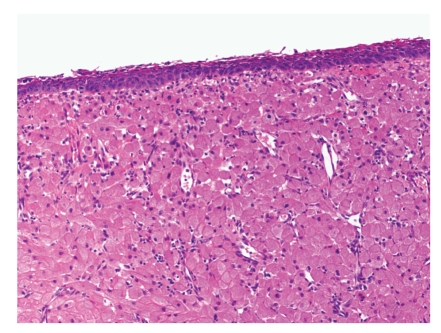
Hematoxylin and eosin stain ×100 showing overlying stratified sqamous epithelium and vascular stroma.

**Figure 4:**
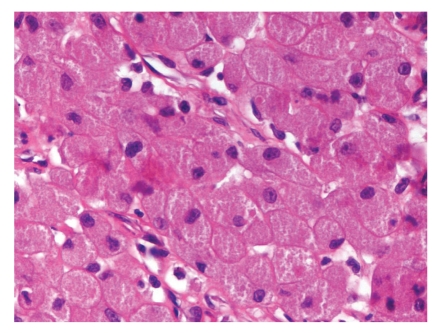
Hematoxylin and eosin stain ×400 showing clusters of cells containing abundant granular eosinophlic cytoplasm and small uninform nuclei.

The two masses were fixed and examined histologically (Figures 3 and 4). They showed sheets and clusters of cells containing abundant granular eosinophlic cytoplasm and small uninform nuclei, along with some myxoid areas and areas of haemorrhage and ulceration, confirming the diagnosis of Congenital Epulis.

## 3. Discussion

Congenital Epulis is a rare tumour of the neonate. Zucker and Buenecha [5] found only 167 cases reported before 1993. It commonly presents in the neonate, although prenatal diagnosis with Ultrasound has been reported as early as 26 weeks gestation [6]. The lesion usually arises over the incisor-canine region of the maxilla (maxillary/mandibular ratio 3:1) [7]. Simultaneous involvement of both maxillary and mandibular alveolar ridges occurs in approximately 10% of reported cases [8]. The lesions commonly interfere with feeding. The diagnosis is usually made on clinical grounds alone, although difficulties may arise when the size of the lesion is small, or the index of suspicion is low. The postnatal ultrasound and MRI appearances of Congenital Epulis have been described. MRI is useful for diagnosis, and superior to ultrasound, showing the gingival origin of Congenital Epulis without local extension [9]. Treatment is with surgical excision, although spontaneous regression has been reported [10].

Epulis is a Greek term literally meaning “of the gums” and is used to describe a wide variety of gum lesions, regardless of their pathological origin. Histologically, Congenial Epulis shows remarkable similarity with the more common Granular Cell Tumours (GCTs) [2, 11]. There are, however, many distinguishing features, such as occurrence solely in the neonate, typical location, plexiform arrangement of capillaries, and lack of pseudoepitheliomatous hyperplasia [12]. GCTs are ubiquitous neoplasms occurring in all age groups, very rarely affecting the gingiva, and can occasionally show malignant change. Immunohistochemical studies have revealed further differences, demonstrating the reactivity of GCTs to S-100 protein and laminin, and their absence in Congenital Epulis [11]. Vered et al. [13] have also recently expanded the immunophenotypic distinction between the two, showing GCTs stain positive for NGFR/p75 and inhibin-*α*, whereas Congenital Epulis does not.

The precise origin of Congenital Epulis remains unclear. CGTs are considered to arise from Schwann Cells, and hence show strong reactivity to S-100 protein [2]. Various theories of the origin of Congenital Epulis include myoblastic, neurogenic, odontogenic, fibroblastic, and histocytic [3]. Lack et al. believe it to be basically reactive in origin [14]. It has been suggested that the occurrence of Congenital Epulis solely in neonates, and more commonly in females, implies a hormonal mechanism of development. However, numerous reports have shown no evidence of either oestrogen or progesterone receptors, and as such suggest an alternative histogenesis [11, 14]. In a review of 33 lesions, Vered et al. conclude that the immunohistochemical profile does not imply any specific cell types for the histogenetic origin of Congenital Epulis [13].

No estimation of incidence of Congenital Epulis has been made to date, to the best of our knowledge. One centre in the USA saw only two cases over the period of 21 years [15]. In University Hospital of Wales, a tertiary referral centre for Otolaryngology and Neonatology, this is the only recorded case of Congenital Epulis since 1980, a total of 28 years. Using incidence of live births (157,454) within that time period, we calculate an incidence of 0.0006% (upper 95% confidence interval: 0.0035%, the inverse of the cumulative Beta distribution [16]). Although most likely an underestimate, this calculation will serve as an approximation of incidence before a more thorough estimation can be undertaken.
